# A Hierarchical Path Planning Approach with Multi-SARSA Based on Topological Map

**DOI:** 10.3390/s22062367

**Published:** 2022-03-18

**Authors:** Shiguang Wen, Yufan Jiang, Ben Cui, Ke Gao, Fei Wang

**Affiliations:** 1 Faculty of Robot Science and Engineering, Northeastern University, Shenyang 110169, China; wenshiguang@mail.neu.edu.cn (S.W.); 1901937@stu.neu.edu.cn (B.C.); 2 College of Information Science and Engineering, Northeastern University, Shenyang 110819, China; 2000739@stu.neu.edu.cn

**Keywords:** hierarchical path planning, mobile robots, topological maps, reinforcement learning

## Abstract

In this paper, a novel path planning algorithm with Reinforcement Learning is proposed based on the topological map. The proposed algorithm has a two-level structure. At the first level, the proposed method generates the topological area using the region dynamic growth algorithm based on the grid map. In the next level, the Multi-SARSA method divided into two layers is applied to find a near-optimal global planning path, in which the artificial potential field method, first of all, is used to initialize the first Q table for faster learning speed, and then the second Q table is initialized with the connected domain obtained by topological map, which provides the prior information. A combination of the two algorithms makes the algorithm easier to converge. Simulation experiments for path planning have been executed. The results indicate that the method proposed in this paper can find the optimal path with a shorter path length, which demonstrates the effectiveness of the presented method.

## 1. Introduction

For decades, mobile robots have remained as one of the most popular research fields and are receiving more and more interest [1,2]. Motion planning is amongst the most basic issues of mobile robot navigation in a strange environment [1], which consists of path planning and trajectory planning. Path planning is a strategy for composing a path, and the sequence of points or curves connecting the starting and ending positions is called a path. Path planning algorithms have been continuously proposed for enhancing the autonomy of the mobile robot in complicated environments [1]. Nowadays, research on motion planning is in a period of vigorous development [3].

Humans can move easily, even in an unknown environment, but it becomes extremely complicated for robots. When people enter an unfamiliar environment, some common knowledge is always used subconsciously for reaching where people want to go [4]. For example, take the kitchen as the target. The dining room is connected to the kitchen instead of the toilet based on our existing knowledge, in which the dining room is a regional representative that helps us narrow the scope of exploration and determine the direction of the target region. Although this human behavior pattern is not necessarily the best way, it must be considered an excellent way in all the knowledge people know. The planning efficiency can be enhanced accordingly if the robots can think like a human in these planning works [4].

The robot can be trained by prior knowledge similar to human common knowledge about the relationship between regions that can be obtained in form of a topological map [4]. Then, the robot sequentially selects the next sub-target based on the topological map which describes the adjacency of a free area [4]. Reinforcement learning (RL), a crucial category of machine learning methods (ML), has increasingly obtained attention in recent years, which can solve the problem of sequential decision making [2,5,6]. Furthermore, RL, unlike the majority of ML methods, has inherent advantages in path planning because of the unsupervised active learning method [7,8]. Not only can it effectively avoid obstacles, but it can also generate the optimal path through numerous experiments in an unknown environment [1].

It is a wise choice to introduce reinforcement learning in path planning, but in the single reinforcement-learning-based path planning algorithm, there exists a problem that the learning sample set is too large while traditional methods are prone to fall into local optimum and need to adjust the parameters. Therefore, a hierarchical path planning method using reinforcement learning is proposed in order to solve the above problem, which aims to spend the minimal computational cost to find a collision-free path, while ensuring the practicality and superiority of the path. First of all, the algorithm deal with the grid map to generate a topological map for dividing the explored environment in the first level. After which, the nodes and adjacencies of the topological map are input into the next layer. In the second level, the Multi-SARSA algorithm is designed for finding a near-optimal global planning strategy. To begin with, the Multi-SARSA algorithm uses the artificial potential field method to modify the Q-table to speed up the learning. Moreover, it scans the adjacency relationship of the topological nodes and initializes the Q-table with the connected domain, which can promote the convergence of the algorithm quickly.

The rest of this paper is organized as follows: In Section 2, we introduce some related work; then, the Multi-SARSA method based on the topological map will be introduced in Section 3. Section 4 shows the simulation results and comparisons and discusses the proposed method. We summarize our contributions and provide a discussion of future work in Section 5.

## 2. Related Works

Path planning is critical for autonomous mobile robots [1]. A variety of methods have been continuously proposed. In the early days, most path planning works were based on geometric methods, such as the visibility graph method and the artificial potential field method [2,9,10].

Recently, for improving the efficiency of path planning, sampling-based algorithms have been purposed [1]. Among them, the Rapidly Exploring Random Tree (RRT) [11] algorithm was put forward as a random sampling tree structure planning algorithm in 1998 and carried out in various path planning works. Because the path found by the sampling method is not necessarily the optimal path, intelligent path planning methods appeared, which include path planning based on fuzzy logic, on genetic algorithms and on neural networks. However, many intelligent methods use supervised learning that requires supervised examples to train mobile robots, which is difficult to obtain in reality [1]. Hence, the reinforcement learning method has been proposed, which is used as the primary and alternative method beyond traditional methods. So far, many reinforcement learning algorithms have been applied to robotics [2,7,9,12,13,14,15]. For example, Q-learning [16] and Sarsa [17] proposed by Watikins, which can handle discrete state spaces well. Although a lot of research work has been conducted in this field, generating the best path in a complex environment is still a challenge for the community.

As the number of obstacles and the complexity of the shape of obstacles increases, planning methods based on hierarchical structure have been gradually proposed to advance the quality of paths in complicated environments [1]. The main advantage of hierarchical methods is its computationally efficient and feasibility. Rösmann. C et al. [18] proposed a online trajectory planner, which mainly focuses on operating in the subregion environment. However, the method keeps the cost to a minimum in the subregion and does not adapt to global planning and navigation. In [19], a hierarchical path planning method is proposed that searches paths by traversing the hierarchy based on the Dijkstra algorithm. Zuo and Guo [1] proposed a hierarchical path planning method in which it uses the grid-based A* algorithm to quickly find the geometric path in the beginning. After which, it uses Least Squares Strategy Iteration to obtain near-optimal local planning strategies. However, the regular grid search is too local, and its applicability to the environment is limited. The work in [9] proposed an algorithm that uses dynamic search factor technology to optimize the traditional Q-Learning method, but the generalization ability needs to be enhanced. The work of Yu song combined the artificial potential field method with reinforcement learning, which, to a certain extent, overcomes the defect of the classic artificial potential field method. However, it is still limited by oversized search space. A hierarchical approximate cell decomposition method was presented for path planning in [20], which is based on the Dubins’ theorem to simplify the problem and searches the path based on the genetic algorithm. The approach proposed in [21] is a hierarchical approximate cell decomposition, which decomposes the configuration space of the robot into rectangloid cells at successive levels of approximation. However, this method of dividing into approximate rectangles is more dependent on the geometry of the configuration environment. The method performs top-down segmentation and finds the sequence of contiguous empty cells which connect the robot’s initial configuration and target configuration. If no such sequence is found, it can only decompose the cell into smaller cells and search again, which not only consumes resources but also leads to the adaptability of the algorithm changing with the geometry of the environment. A layered goal-oriented motion planning strategy is proposed in [22], which uses fuzzy logic to produce an intermediate goal in the first layer and uses short-range sensory data in the second layer to guide the robot to reach it. However, its performance is strongly dependent on the sophisticated sensory systems, such as sonar sensors and GPS. An approach to automatic path planning based on a quadtree representation is presented in [23], in which hierarchical path-searching methods are introduced. However, quadtree representation of free space cannot localize the effect of obstacles, which is a general shortcoming of cutting free space into rectanguloid cells.

Inspired by the above methods, a hierarchical path planning method purposed with Muti-SARSA based on topological maps to narrow the search space and quickly search the optimal or near-optimal path.

## 3. Proposed Methodology

The problem addressed in this paper is to perform path planning with topological maps generated by grid maps. This work provides a hierarchical path planning algorithm that can be applied to any environment, regardless of the geometric structure of the environment. The mathematical model of path optimization is presented in this part briefly. After which, the approach which is based on the Multi-SARSA algorithm and topological map will be introduced in detail.

### 3.1. Mathematical Model of Path Optimization

**Figure 1:**
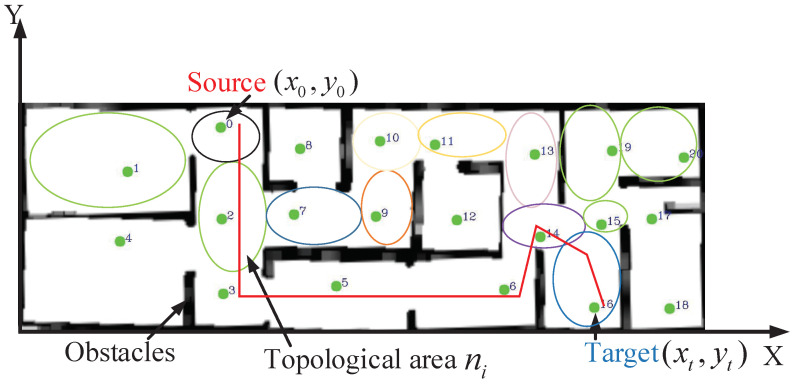
The schematic diagram of path planning mathematical model based on topological map. In the figure, $\left(x_{0},y_{0}\right)$ is the starting point, $\left(x_{t},y_{t}\right)$ is the the end point and the green points 1–18 are the topological nodes representing the topological areas.

Path planning is to search a path consist of a set of points that is collision-free and feasible for robots moving from the start position $S_{0}=\left(x_{0},y_{0}\right)$ to the target position $S_{t}=\left(x_{t},y_{t}\right)$ [1]. The generated path macroscopically consists of topological areas represented by topological nodes $\left(N_{i},i=1,\cdots ,n\right)$. To suppose a robot that explores in an environment with *n* topological nodes, in which whole information about topological regions is obtained. Every topology area corresponds to a topology node $P_{i}=\left(x_{i},y_{i}\right)$ as shown in Figure 1. The state of the mobile robot in this paper is defined as $S_{i}=\left[x_{i},y_{i},K\right]$ under the global coordinate, where $x_{i}$ and $y_{i}$ are the two-dimensional coordinates of the robot in the grid map which convert according to the current position of the robot (CurrentPos). *K* represents the topological area to which the current coordinates belong, which updates only when the topological area that the current coordinates belong to changes. The mathematical equation of the path planning is given as follows:

(1) $$R\left(S_{0},S_{t}\right)=Min\left\{Step_{N_{c}}^{N_{h}}\right\},\;\left\{\begin{array}{c} N_{c}\in N_{i},i=1,2,\cdots ,n\;\;,\\ N_{h}\in C_{c},\\ S_{i}\notin obstacle,\\ S_{i}\neq S_{i+1},\\ N_{c}\neq N_{h} \end{array}\right.$$

where:

*n*: Number of topological nodes generated by the grid map;
$N_{i},i\in \left[1,n\right]$: Node name;
$S_{c}$: The current position of the robot;
$N_{c}$: Topology area that the current position belongs to;
$N_{h},N_{h}\in C_{c}$: Candidate node;
$C_{c}=\left\{N_{i}|N_{i}\neq N_{c},i=1,3,\cdots \right\}$: Connected domain;
$Step_{N_{c}}^{N_{h}}$: Number of steps required from the current topology area to the candidate topology area. Here, the candidate topology node cannot be changed before reaching the candidate topology area, and the key decision condition in changing *K* is that robots only need to reach the candidate topology area instead of the candidate topology node.

### 3.2. The Framework of the Proposed Method

The single reinforcement learning-based path planning algorithm exists the problem that learning sample set is too large. In order to solve the problem, the proposed approach hence is divided into two layers. In the first layer, the pre-given topological nodes is set as the prior information to narrow the set of samples to be searched, in which the algorithm first chooses macro-level topology nodes by reinforcement learning (which is equivalent to the global path planning) and then selects the optimal candidate region according to the topology relationship. The method in this way avoids global search and narrows the search range. The algorithm will be explained in detail in Section 3.3.

After which, the nodes and adjacencies of the topological map are input into the next layer. The second layer performs path planning with inter-mediate nodes (equivalent to local path planning), in which the Multi-SARSA algorithm is used for finding a near-optimal global planning strategy.

**Figure 2:**
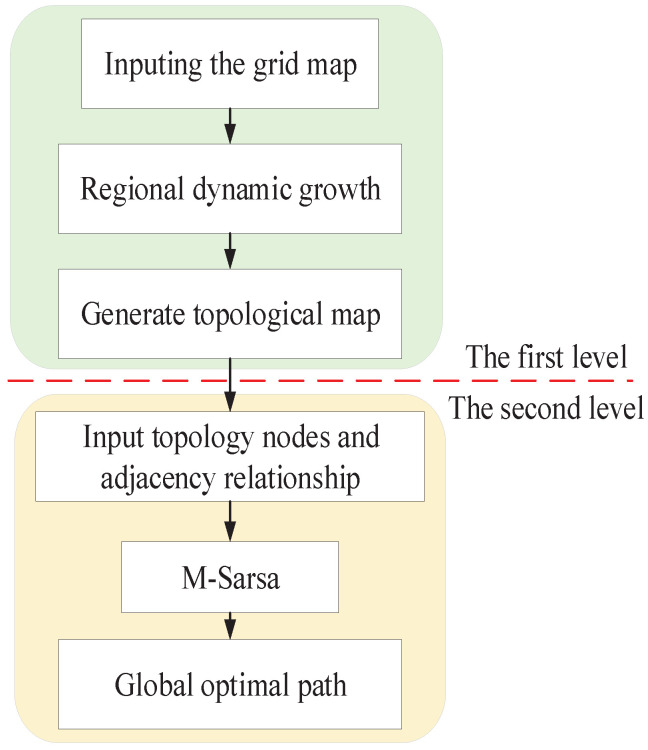
The framework of the proposed method.

The Multi-SARSA algorithm divided into two layers is applied after obtaining a topological map to search a near-optimal path, which is based on the artificial potential field method to modify the Q-table in the first layer for reducing the blindness in the exploration. After which, so as to accelerate the training efficiency and convergence rate, the adjacency relationship of the topological nodes is conducted as the prior knowledge to initialize the second Q-table. In this way, instead of randomly traversing all the maps during learning, robots only traverse the current area and the candidate area connected with the current node based on the prior information, which limits the search space. Figure 2 demonstrates the framework of the proposed path planning algorithm.

### 3.3. Generate a Topological Map

In a grid map, the geometric structure of complex cluttered environments can be divided into several barrier-free geometric shapes as indicated in Figure 1, which can represent the vacancy area and contain connectivity information simultaneously.

Use the region growth method in Wang et al. [24] to construct the topological map, whose main process is the following: After the grid map is obtained, because the trajectory where the robot moves must be free, the growth point is randomly selected on the driving trajectory; “Then grows dynamically according to the parameters until the regional characteristics meet the requirements, the growth will stop. After the growth of each region is completed, it will be segmented from the grid map, and the color identification of the RGB topological will be established” [24]. The gravity center of the topological areas will be set as topological nodes while the neighborhood is scanned [24]. More details on these steps can be found in the previous work [24].

**Figure 3:**
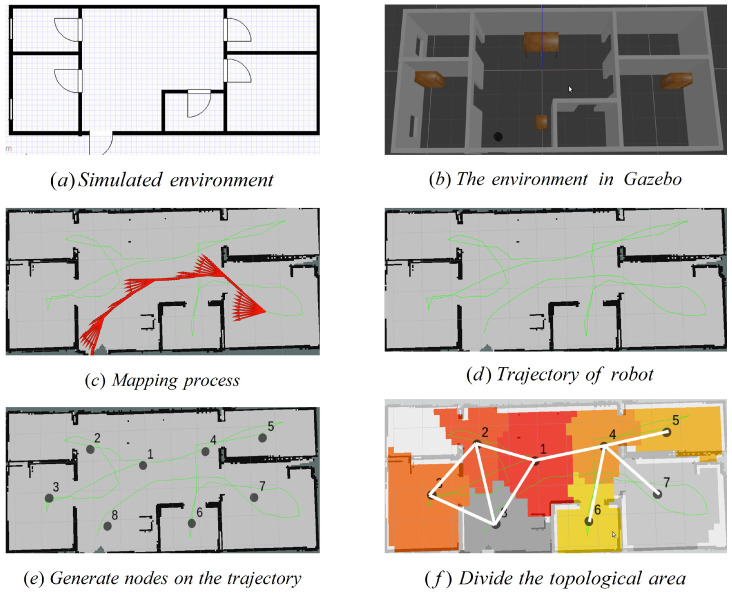
The steps of topological map generation in a simulated environment.

The simple simulation environment is built on Gazebo, and a topological map is generated of this environment as shown in Figure 3. Figure 3a is the design drawing of the simulated environment and Figure 3b illustrates the simulation environment. Figure 3c is the exploration process of the robot generating a raster map, in which the green line is the exploration trajectory of the robot and the red arrow is the odometer information. Figure 3d describes the final generated raster map. Figure 3e is the topology node generated in the map and the topological map example is shown in Figure 3f.

### 3.4. Multi-SARSA Algorithm

#### 3.4.1. Classical Sarsa Algorithm and Its Limitations

Reinforcement Learning, RL, is one of the paradigms and methodologies of machine learning, in which State-Action-Reward-State-Action(SARSA) [17], as an algorithm for learning Markov decision-making process strategies, is usually applied in the field of machine learning and reinforcement learning. The core idea of the algorithm can be simplified as:

(2) $$Q\left(s_{t},a_{t}\right)\leftarrow Q\left(s_{t},a_{t}\right)+\alpha \left[r_{t}+\gamma Q\left(s_{t+1},a_{t+1}\right)-Q\left(s_{t},a_{t}\right)\right]$$

where α is the learning rate, which determines the extent that information newly acquired covers old information. γ is the discount factor, which determines the importance of future rewards. When γ is 0, the agent will become “opportunistic” and only consider the current reward. When γ is close to 1, the agent will strive for long-term high returns. $r_{t}$ represents the reward/penalty that the agent receives after performing the action.

The time SARSA algorithm required to optimize the path will increase when the search space increases, which prevents the algorithm from converging to a better path.

#### 3.4.2. Multi-SARSA Algorithm

**Figure 4:**
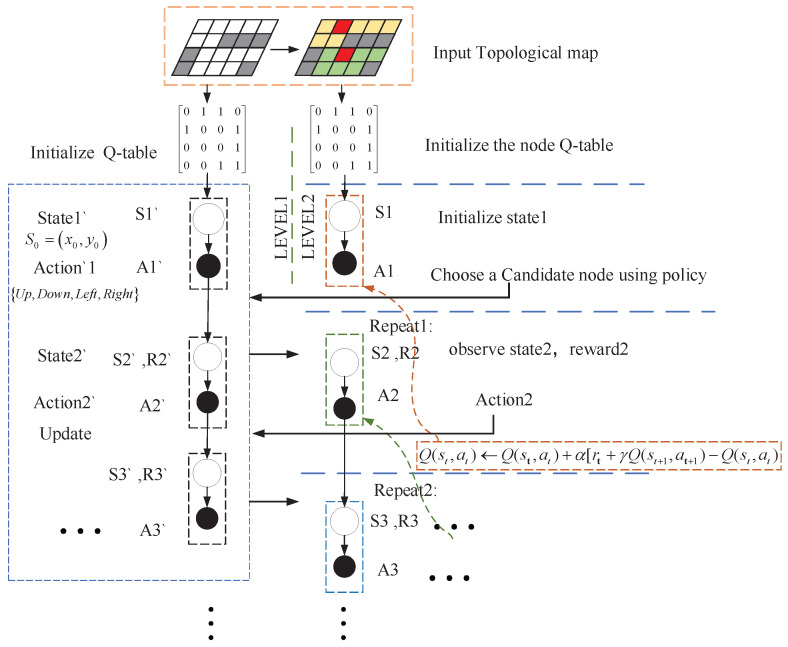
The framework of Multi-SARSA algorithm structurally improved, in which the left column is the first layer and the right column is the second layer, and the a priori information is provided by the topological map.

The Multi-SARSA algorithm is proposed based on the original SARSA algorithm, in which a simple improvement in the structure was implemented to make the algorithm easier to converge. The overall framework of Multi-SARSA is shown in Figure 4.

The initialization method proposed in this article draws on the idea of the artificial potential field method. In the beginning, the algorithm takes the topological map obtained in section C as the input, which, as the prior information of the algorithm, includes topological areas and connected domain information.

At the beginning of exploration, the agent is moving randomly, which leads to wastage of computational resources and limit to converge. To accelerate the learning process, the artificial potential field method is used to initialize the first-level Q-table of Multi-SARSA.

The artificial potential field method [10], in simple terms, is setting obstacles as repulsive forces and targets as attractive forces, adding force vectors, and, finally, calculating the direction of the resultant force and using the resultant force received by the robot in a virtual potential field environment to determine which direction of the next step. The attractive potential function $U_{att}\left(s_{i}\right)$ is:

(3) $$U_{att}\left(s_{i}\right)=\frac{1}{2}\xi \rho^{2}\left(s_{i},s_{t}\right)$$

where $s_{i}=\left(x_{i},y_{i}\right)$ is the current position, $s_{t}=\left(x_{t},y_{t}\right)$ is the target position, ξ is the gravitational gain factor, and $\rho (s_{i},s_{t})$ indicates the distance between the current state and target. Gravity is the derivative of the gravitational field with respect to distance:

(4) $$F_{s_{t}}\left(s_{i}\right)=-\nabla U_{att}\left(s_{i}\right)=\xi \left(s_{d}-s_{i}\right)$$

In the traditional repulsion field, the repulsive potential function $U_{rep}\left(s_{i}\right)$ is:

(5) $$U_{rep}\left(s_{i}\right)=\left\{\begin{array}{c} \frac{1}{2}\eta {\left(\frac{1}{\rho (s_{i},s_{o})}-\frac{1}{\rho_{0}}\right)}^{2}\;\;,\;\;if\rho \left(s_{i},s_{o}\right)\leq \rho_{0}\\ 0\;\;\;\;\;\;\;\;\;\;\;\;\;\;\;\;\;\;\;\;\;\;\;\;\;\;\;\;\;\;\;\;\;\;\;\;\;\;\;\;\;\;\;\;\;\;\;\;\;\;\;\;\;\;\;\;\;\;\;\;\;\;\;\;\;\;\;\;\;\;\;\;\;,\;if\;\;\rho \left(s_{i},s_{o}\right)>\rho_{0} \end{array}\right.$$

where η is the repulsive force scale factor, and $\rho \left(s_{i},s_{o}\right)$ represents the distance between robots and obstacles. $\rho_{0}$ represents the influence radius of each obstacle, which means that the obstacle will have no repulsive effect when the distance is longer than the set radius. The total field is the superposition of the repulsive field and the attractive field, which is:

(6) $$U\left(s_{i}\right)=U_{att}\left(s_{i}\right)+U_{rep}\left(s_{i}\right)$$

(7) $$F\left(s_{i}\right)=-\nabla U\left(s_{i}\right)$$

**Figure 5:**
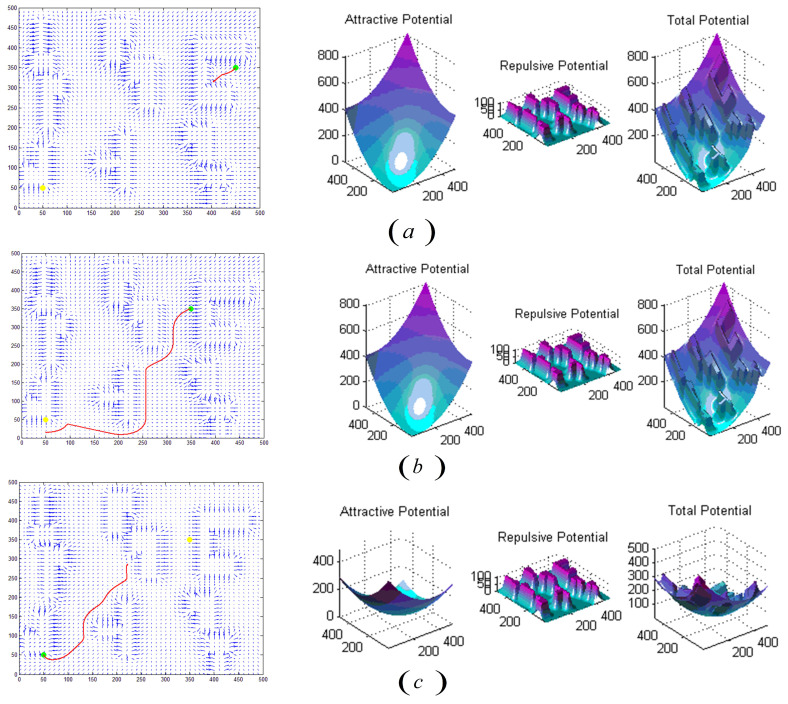
The path planning results of artificial potential field method on MATLAB: (**a**) Set the green dot in the upper right corner as the starting point and the yellow dot in the lower left corner as the end point, while the three-dimensional graphs of the attractive field, repulsive force field, and the total force field on the right side. Compared with (**a**,**b**) moved the starting point, and (**c**) exchanged the starting point and the end point. The result is shown in the figure.

However, the problem of the purely artificial potential field method is that there may be a local optimal region in an unknown obstacle environment, which will cause the exploration method to fall into the local optimal region, thus leading to failure. For example, the situation is shown in Figure 5. The planned path is difficult to approach the start/endpoint when its location is too close to the obstacle, the final planning thus fails, or the path becomes stuck in a dead-end during the gradient descent process when the starting point and ending point are set as shown in Figure 5, thus failure. Therefore, this section provides a strategy to improve the algorithm. The key idea in solving the local optimal problem is using the artificial potential field method to initializes the Q table, which provides a prior experience of the working space for the robot.

In the method, the potential field is normally built depending on the Euclidean distance from each position to the target position, which, mathematically speaking, is a gradient descent process [9]. In this article, since robots, in SARSA, move from a low Q-value position to a high Q-value position, the inverse field force is thus used to update the Q table.

**Figure 6:**
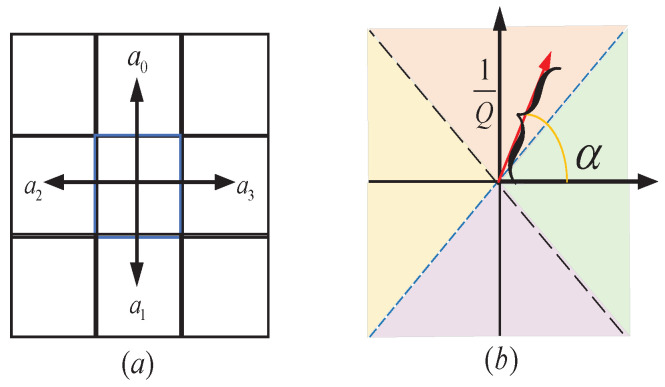
(**a**) The four directions that the robot can move corresponding to four actions. (**b**) The way to convert angles to actions, which 360 degrees are divided into four sections, that is, −ππ44,ππ44], [π/4, 3π/4], [3π/4, −3π/4], [−π/4, −3π/4].

Assuming that the robot moves at a fixed speed, the robot can be regarded as a point in the experiment [9]. In order to reduce the path distance and direction angle of the robot, four actions are utilized in this article. As shown in Figure 6a, action 1, 2, 3, and 4 denote the up, down, left, and right, respectively.

Then, the action is selected according to the angle of the resultant force, that is, the front, back, left, and right actions are divided by the angle of the axis. According to the angle range to which the deflection angle belongs to, the specific movement direction is determined, and the corresponding Q-value is updated. The angle margin division is shown in Figure 6b. The robot chooses the next action according to the Q-value of the surrounding 4 neighboring grids with a 90% probability of transferring to the grid which action has the largest Q-value and a 10% probability of moving one step randomly in the direction of the surrounding 4 grids.

A separate example simulation experiment was carried out for this part in MATLAB, and the effect diagram is as follows. Taking the experimental data as an example, the Q table can be initialized according to the angle as shown in Table 1. The relevant pseudo-code is in Algorithm 1. This method initializing the first-level Q table provides a priori experience of the workspace for the robot and avoids lack of information at the beginning of learning, thus accelerating convergence.

**Table 1:** The experimental results corresponding to Figure 7 and the results of initialization according to Figure 6.

Step	Force Size	Force Angle	Front	Back	Left	Right
1	106	0.7854	0	0	0	0.00943396
2	111.6930	0.7933	0	0	0	0.00895311
3	132.7176	0.8138	0.00753479	0	0	0
4	184.0985	0.8475	0.00543187	0	0	0
5	320.2240	0.8909	0.003125	0	0	0
6	100.4174	80.3598	0	0	0	0.008620
…	…	…	…	…	…	…
36	13.3221	1.1384	0.075	0	0	0
37	8.8253	1.2403	0.1133	0	0	0
38	5.3136	1.3531	0.1882	0	0	0
39	2.0616	1.4441	0.4506	0	0	0

**Algorithm 1** Artificial Potential Field Method.**Input:** Obstacle $p_{o}\left(x_{i},y_{i}\right)$, Gravitational gain coefficient ξ,η, Obstacle influence distance $p_{0}$, starting point $S_{0}$, endpoint $S_{t}$, Max number loops *J*.**Output:** Resultant value *F*, Resultant value α, step *n*.1:**for**i=1 to *J* **do**2:    Calculate Attract Module ($S_{i},S_{t},\xi ,angle\;\_\;att$)3:$\begin{array}{c} \;\;d=sqrt\left({\left(S_{i}.x-S_{t}.x\right)}^{2}+{\left(S_{i}.y-S_{t}.y\right)}^{2}\right)\\ \;\;\left[f_{1}.x,f_{1}.y\right]=\left[\xi \cdot d\cdot \cos(angle\;\_\;att),\xi \cdot d\cdot \sin(angle\;\_\;att)\right] \end{array}$4:    Calculate repulsion module ($S_{i},S_{o},\eta ,angle\;\_\;rep,p_{0}$)5:          $f_{2}^{rep}.x,f_{2}^{rep}.y,f_{2}^{att}.x,f_{2}^{att}.y$6:    Calculate the resultant force:7:$\begin{array}{c} \;\;F.x=f_{1}.x+f_{2}^{rep}.x+f_{2}^{att}.x\\ \;\;F.y=f_{1}.y+f_{2}^{rep}.y+f_{2}^{att}.y \end{array}$8:            $angle=a\tan(F_{y}/F_{x})$9: 10:    If (arrive terminal)11:          Print *n*; Break;12:**end for**13:**return***n*, angle

##### The Second Level

The heuristic search strategy needs to be combined with greedy search to jump out of the local optimal region. To further accelerate the convergence, a topological map-based search algorithm inspired by the greedy search is designed for the second layer. The algorithm provides selectable candidate areas based on the topological map and passes them to the first level as the sub-objective. After that, the first level generates an achievable local optimal path for robots to achieve them sequentially. The advantages of hierarchical planning methods are efficient computation and simple to implement, which makes the algorithm more robust [1].

The essential point of the proposed search method is primarily determining the topological area of the current robot according to the state of the robot and then finding the area connected to the current topological node according to the prior information. After which, the closer node to the end-point is selected as a candidate topology node according to the strategy. After selecting the exploration area, the algorithm stipulates that only the topological area and the candidate topological area connected by the current robot state are explored in the learning process. Then, the first-level algorithm is used to reach the candidate child nodes. In the topological map search algorithm, the judgment condition to end the learning is as long as the candidate area is reached, which is equivalent to reaching the corresponding candidate nodes. The algorithm speeds up the learning time by reducing the search space in this way, which angle judgment conditions are the same as above and the state and action definitions are shown in Table 2.

**Figure 8:**
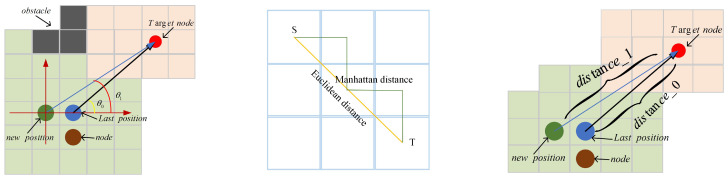
The (**left picture**) is an angle diagram inspired by DWA, and $\theta_{1}-\theta_{0}$ in the picture is the angle difference needed; the (**middle picture**) is a comparison diagram of Euclidean distance and Manhattan distance; and the (**right picture**) is a distance diagram, in which diatance_1−distance_0 is diatance(v,w) in the function.

In the current node, several sets of actions are feasible to reach the next target node, and the reward function thus is used to evaluate every action. The reward distribution procedure is invariably approaching the child node with the shortest distance within the specified area, which helps to enhance the option of the best action. Through the reward distribution algorithm to explore a specific area, let the robot move to the fastest target point around the currently connected area and candidate area in each learning step, so as to obtain the best path. Inspired by the DWA (dynamic window approach) algorithm [25], the reward function designed in this paper is as follows:

(8) G(v,w)=σ(α·angle(v,w)+β·distance(v,w))

where angle(v,w) is the azimuth angle evaluation function, which is used to evaluate the angle difference, that is, $\theta_{1}-\theta_{0}$, as shown in Figure 8. In this paper, the method of 180−θ is used to evaluate, which means the smaller the value, the higher the evaluation score. distance(v,w) is applied to evaluate the distance between the current position and the target node, for which realization method is to compare the distance between the current position and the target node with the Euclidean distance from the previous position to the target node. Then, the reward value is given according to the comparison result, that is: distance_1−distance_0.

Finally, smoothing and normalization are performed. The formula is as follows:

(9) $$\begin{array}{c} normal\;\_\;angle\left(i\right)\;\;=\;\;\frac{angle(i)}{\sum_{i=1}^{n}angle(i)}\\ normal\;\_\;distance\left(i\right)\;\;=\;\;\frac{distance(i)}{\sum_{i=1}^{n}distance(i)} \end{array}$$

The algorithm pseudo-code is in Algorithm 2.
**Algorithm 2** Multi-SARSA Algorithm.1:Initialize $Q\left(s,a\right)$ based on Topological node2:      Repeat (for each episode) connectivity3:            Initialize *s*4:            Choose a Candidate node from s using derived from *Q*5:            Return the first level6:            Repeat (for each step of episode)7:                  Take action $a^{\prime }$, observe $r,s^{\prime }$8:                  Choose $a_{next}^{\prime }$ from $s^{\prime }$ using policy derived from $Q^{\prime }$9:            Until $s^{\prime }$ is Candidate area

## 4. Experiments

Experiments are conducted in a simulated environment running on the Gazebo platform to indicate the effectiveness of the presented approach in this paper. The software systems are carried out in Robot Operation System(ROS) on top of Ubuntu 16.04 LTS. About the reinforcement learning part, the 25 × 25 grid maze world is built in the simulation environment, in which the start point, obstacle, and end point are marked. Then, the effectiveness of the algorithm is verified on the DELL Precision T7.

An array of experiments are executed to generate the planning path. Particularly, according to the category, the experimental methods include geometric methods, search methods, and reinforcement learning methods.

### 4.1. The Geometric Methods: The Artificial Potential Field Method

**Figure 9:**
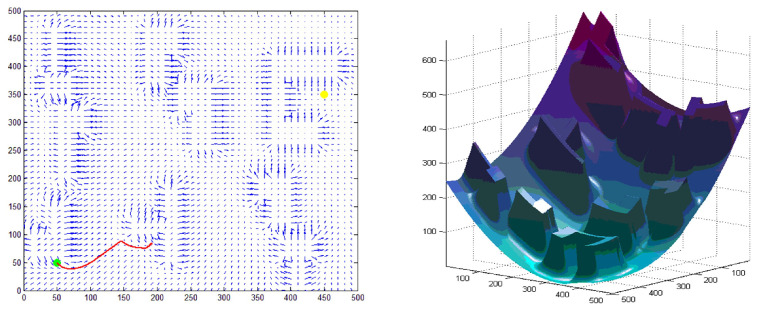
Experimental results of the artificial potential field method under similar scenes and the same starting point and end point settings (failed).

In the geometric method, an experiment using the artificial potential field method has conducted on MATLAB. The algorithm regards the target and obstacles as objects that have attractive and repulsive forces to the robot, respectively. Robots move along the total force of the attractive and repulsive forces. The total potential field combining the attractive potential and the resultant potential is shown in Figure 9.

According to the gradient display of MATLAB, as shown in Figure 9, the green dot is set as the starting point and the yellow dot is the target point. Each small arrow in the figure represents the size and direction of the attractive forces at the current position. The gradient of position far away from the obstacle obviously points to the yellow endpoint. The closer the endpoint is, the smaller the gradient value; the gradient closer to the obstacle shows a tendency of repulsion, that is, a shorter distance from the obstacle results in a greater repulsion force.

In Figure 9, the method falls into a local optimal solution, which leads to planning failure.

### 4.2. Search Algorithms: A* and Iterative Deepening A*

In the search algorithm, the A* [26] algorithm is amongst the most prevalent heuristic search algorithms, which can be considered as an extension of Dijkstra’s algorithm.

**Figure 10:**
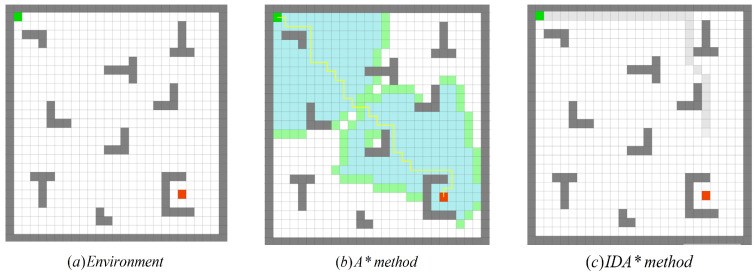
The experimental results of the search algorithm in a similar environment: (**a**) the experimental environment; (**b**) the planning result of the A* algorithm based on Euclidean distance (success); (**c**) the planning of the IDA* algorithm also based on the Euclidean distance result (failure).

The autonomous exploration process in a typical simulation environment is shown in Figure 10, in which the green point and the red point represent the beginning point and the endpoint, respectively, while the yellow line is the trajectory of the robot during exploration. The result is depicted in Figure 10b, which indicates that although the plan was successful, the planned path contains too many turning points which can be improved.

The second algorithm chooses the IDA* algorithm [27]. The IDA* algorithm that was proposed by Korf in 1985 is a consolidation of the A* algorithm and the iterative deepening algorithm, whose specific name is the Iterative Deepening A*. The IDA* algorithm is a re-optimization of the A* algorithm.

IDA* experiments were carried out in the same environment. However, the experimental results as shown in Figure 10c indicate that the plan was not successful.

### 4.3. Path Planning Approach Using Reinforcement Learning

#### 4.3.1. Topological Map

Since generating the optimal path in a complex environment is still a challenge for the community, the path planning approach using reinforcement learning has emerged as the preferred and alternative method beyond traditional methods.

The algorithm in this paper makes a simple structural improvement on the classic reinforcement learning algorithm, SARSA, while combining it with the topological map in terms of initialization. This initialization method can provide prior information and reduce blindness in learning and exploration, thereby speeding up training efficiency.

**Figure 11:**
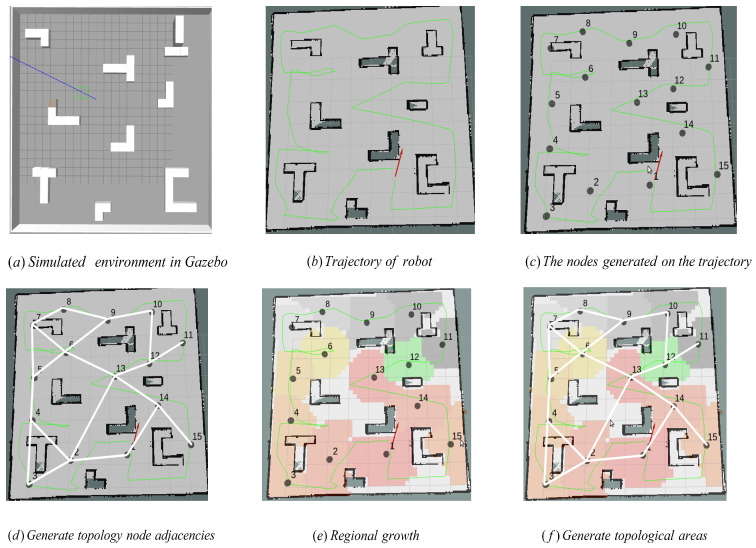
The topological map construction process in a similar environment (basically the same as the process in Figure 3). (**a**) is the simulated environment in Gazebo, (**b**) is the trajectory of robot, (**c**) is the nodes generated on the trajectory, (**d**) is the adjacency, (**e**) is the region dynamic growth algorithm and (**f**) is the Generated topological area.

The simulated experimental environment is shown in Figure 11a, which is similar to the above experimental environment. The picture demonstrated in Figure 11b is the result that uses the gamapping algorithm to map the experimental environment, in which the green line is the trajectory of the car as it explores the environment and the red arrow is its odometer at a certain time. The gray node in Figure 11c is the topological node generated on the driving trajectory of the car according to the regional dynamic growth algorithm, every which can be connected to the Exploration trajectory without meting collision. Figure 11d shows the connectivity of each node and Figure 11e is the topological area represented by each node. The final map is shown in Figure 11f, which contains all the information.

The proposed topological map structure can represent the space topology of the environment appropriately [24], which can be extended to every part of the environment, resulting in outstanding learning.

#### 4.3.2. Multi-SARSA

**Figure 12:**
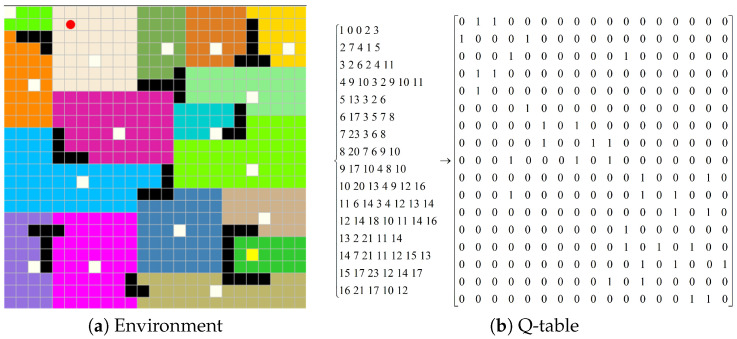
(**a**) The simulation environment generated according to Figure 11 (same as Figure 10a). (**b**) Q-table updated according to the topological relationship in (**a**).

According to the generated topology map, several minor changes are made in order to facilitate comparative experiments. The slightly adjusted environment is shown in Figure 12a, and the modified adjacency relationship corresponding to the simulated environment is as shown in Figure 12b.

The comparison experiment selects the classic Q-learning and SARSA algorithms. The Q-learning algorithm proposed by Watikins is considered as being amongst the most classical RL (Reinforcement Learning) algorithms, the advantage of which is that it is uncomplicated and feasible and the obvious disadvantage is the limited convergence rate.

Several experiments are conducted in a simulated environment, demonstrated in Figure 12a, in which the upper left corner is the starting point and the yellow dot in the lower right corner is the endpoint. The generated colored area is the topological area of the environment. White points in the figures are the representative node of the candidate target regions, which is used to guide the exploration. The robot will head toward to the target regions guided by these blue dots which is the generated planning path.

**Figure 13:**
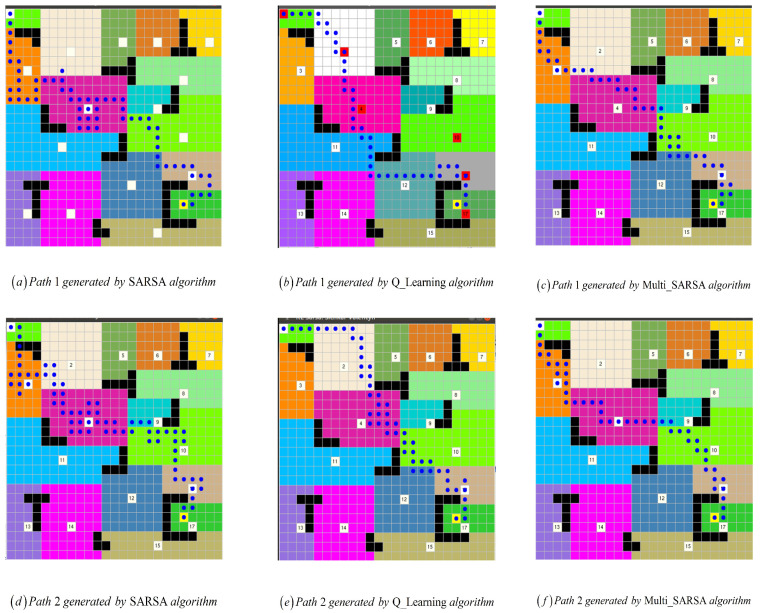
In the same environment, (**a**–**c**) are the planning results of the SARSA algorithm, Q-Learning, and the algorithm proposed in this paper, respectively. The colored part is a topological area, and one color corresponds to a topological area. The white dot is the selected representative topological node, the starting point is the upper left corner, and the end point is set in the lower right corner. (**a**,**d**) are the paths generated by sarsa algorithm, (**b**,**e**) are the paths generated by q-learning algorithm, and (**c**,**f**) are the paths generated by the multi-sarsa algorithm.

**Figure 14:**
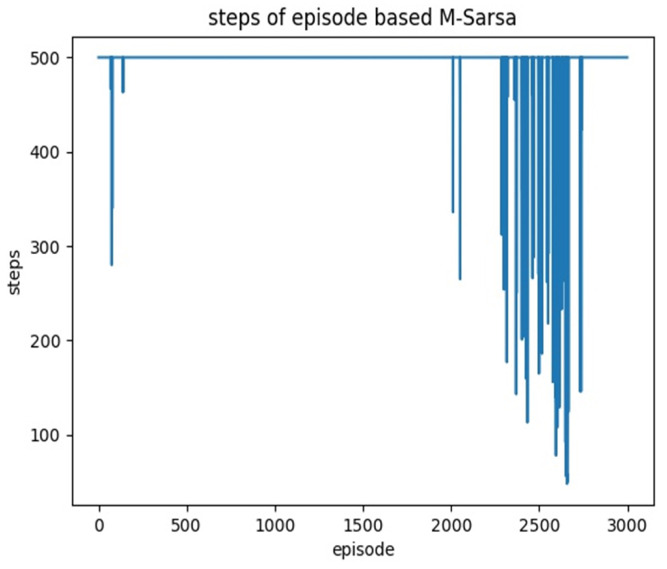
The step diagram of the Multi-SARSA algorithm.

The learning rate and the discount factor are the same and set to 0.01 and 0.9, respectively. It can be concluded from the results as shown in Figure 13a that the effect of SARSA among the three algorithms is the worst. The results of the Q-learning algorithm as shown in Figure 13b are improved compared to SARSA, but the path length and the number of inflection points are not ideal. The M-SARSA proposed in this paper is improved the algorithm on the basis of SARSA; we can see that compared with the original SARSA, our results show that Multi-SARSA, as shown in Figure 13c compared with the Q-learning and A* algorithms, has more advantages in path length and the number of inflection points. Multi-SARSA’s step diagram is shown in Figure 14.

The number of episodes is 3000, and the maximum number of steps per episode is 500. If the agent collides with an obstacle during the movement, it will be considered as a failure in this episode.The corresponding reward will be given, and the number of steps in this episode will be considered as 500. The graph of the experimental results is shown in Figure 14. It can be seen that as the rounds increase, the number of episodes begins to oscillate, indicating that the agent has a good training effect. The greater the oscillation, the better the training effect of the agent. The path diagram is shown in Figure 13c and the results are compared in Table 3.

Qualitatively speaking, the presented method based on a topological map is obviously better than the path planning methods based on SARSA and Q-learning. On the one hand, the proposed topological map is more efficient because of the simplification of the whole environment without resorting to scanning the total environment, which, in this manner, reduces the search space. On the other hand, compared with traditional search algorithms, the use of reinforcement learning makes it easier for the algorithm to obtain a near-optimal path.

#### 4.3.3. Human-like

Furthermore, the good news is that the proposed approach can accomplish human-like planning differently from other approaches, which indicates that the presented approach can plan the path according to the special requirements of each person.

**Figure 15:**
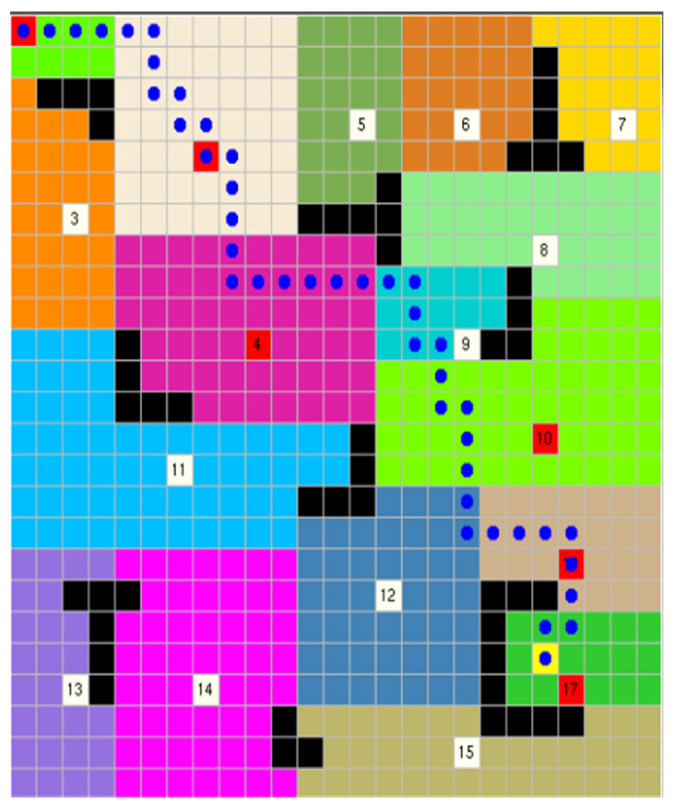
A path planned according to people’s preferences (1-2-4-10-16-17).

When people do not aim to reach the target point as soon as possible, the algorithm can implement it in two lines. It can, according to the requirements of people, not only meet the needs of individuals to reach a certain area but also achieve the terminal as quickly as possible. That allows the algorithm to have a choice space, and human input allows the algorithm to obtain more prior information, which not only greatly enhances the user experience but also further accelerates the process of the algorithm. We also conducted experiments on this part separately, and the experimental results are shown in Figure 15.

This part mainly focuses on providing interface conditions for the work of brain–computer interface, which will be improved in future work.

## 5. Discussion

The single reinforcement-learning-based path planning algorithm has the problem that the search sample set is too large and the traditional methods are prone to fall into local optimum and need to adjust the parameters. In this paper, a hierarchical path planning algorithm based on a topology map is proposed for obtaining the optimal path of a mobile robot with less computational cost. This method divides the complex optimization problem into several simple small problems and solves them one by one in a step-by-step manner. Moreover, the reinforcement learning is used in the hierarchical method to increase the adaptability of the algorithm, which solves the problem of parameter adjustment and reduces the search sample set by giving prior information. Experiments show that, compared to a single algorithm, slightly superior results are achieved with our hierarchical algorithm. At the same time, because of the layering of the algorithm, it is more convenient for people to set staged goals, that is, to introduce more human factors in the operation of the algorithm. This information could be helpful in the application of human–computer interactions. We believe that it will provide new ideas for the application scenarios of the algorithm, such as some application scenarios that pay more attention to comfort, namely, smart wheelchairs.

There are several limitations to this approach. Firstly, there is a preset condition that the method must generate a topology map to provide the a priori information, which creates some restrictions or adds some preconditions for the application scenarios of the algorithm. Nonetheless, we believe that it is well justified to use topological maps, which largely narrows down the search sample set and helps reinforcement learning converge. Secondly, the current experiments are carried out in a static environment. Thus, a study in the dynamic environment is warranted in the future. Thirdly, since a robot in the real world is not a particle but has its real size, the next step of this work will try to add an inflated map to solve this problem based on the existing work. At the same time, future expansion will include expanding application scenarios that the signal of the brain–computer interface is used as the input of the human-like algorithm part, which provides more interactive options for the robot.

## 6. Conclusions and Future Work

In this article, a hierarchical path planning algorithm with Multi-SARSA based on topological maps is presented as a solution to obtain the best path for mobile robots with fewer computing costs.

The method proposed in this paper divides and solves the problem by providing a priori information: The topology map provides child nodes, while the artificial potential field method provides the initialization Q-table. The cooperation of two prior sets of information allows us to accelerate the convergence of the algorithm and provide a better solution.

In order to verify the validity of the method, several experiments have been conducted in the simulation environment. The experiments show that, under the same conditions, the separate artificial potential field method and the IDA* method in the traditional method directly fall into the local optimum and lead to direct failure. The A* algorithm also has different solutions due to different parameters.

Compared with traditional methods, the single reinforcement learning method can complete the path planning, but there can be better solutions. In order to further improve the convergence effect of the algorithm, the proposed algorithm is used on the topological map and the Multi-SARSA algorithm, which optimizes the path by two prior information provided. Compared with the Q-Learning algorithm, the average length of the proposed algorithm decreases by 6%, and the average turning point decreases by 4%. Furthermore, compared with the SARSA algorithm, the improved algorithm based on the original SARSA algorithm has a more obvious improvement, where the average length is reduced by 30% and the average turning point is reduced by 12%. The experimental results show that the method presented in this paper can obtain the near-optimal path with the prior information. The simulation results of the presented approach prove its potentiality and superiority.

The experiment further explores the effect of prior information on reinforcement learning path planning. Experiments show that the presentation information can make the convergence speed of the algorithm faster, and the convergence result is more similar to presentation information, which shows that the proposed method has a good prospect in personalized path training. Therefore, our future work involves developing algorithms on this basis of providing robots with more interactive options.

## Figures and Tables

**Figure 7:**
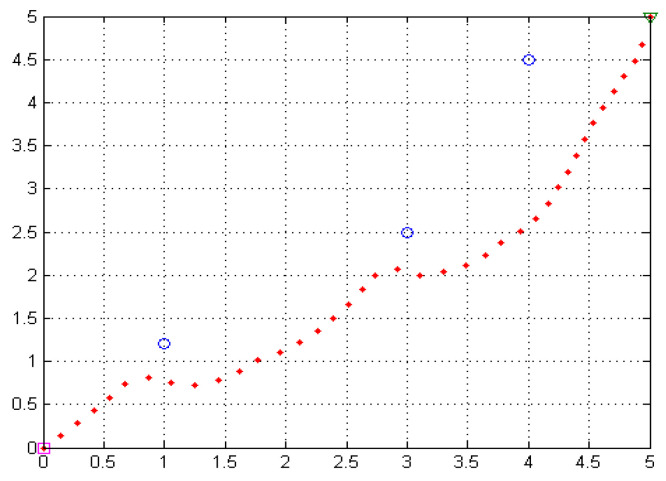
The successfully planned path in MATLAB corresponds to Table 1. The three blue circles in the figure are obstacles, and the red dots are the successfully planned paths.

**Table 2:** The design of the two-layer action and state of the Multi-SARSA algorithm.

	State	Action
Level1	$S_{i}=\left[x_{i},y_{i}\right]$	$\left\{Up,Down,Left,Right\right\}$
Level2	$N_{c}$	$\left\{C_{1},C_{3}\cdots |C_{1},C_{3}\cdots \in N_{i},N_{i}\neq N_{c},i=1,2,\cdots \right\}$

**Table 3:** The experimental results corresponding to Figure 13.

	Average Length	Average Turning Point
SARSA	66	25
Q_Learning	49	23
Multi_SARSA	46	22

## Data Availability

Not applicable.
